# Role of cholecystokinin receptor-A gene polymorphism in development of functional dyspepsia

**DOI:** 10.1186/1755-8166-7-S1-P111

**Published:** 2014-01-21

**Authors:** Rajan Singh, Balraj Mittal, Uday C Ghoshal

**Affiliations:** 1 Departments of Gastroenterology and Genetics, Sanjay Gandhi Postgraduate Institute of Medical Sciences (SGPGIMS), Lucknow-226014, India

## Background

Functional dyspepsia (FD) is characterized by epigastric pain, burning, early satiety and post-prandial fullness in absence of organic or metabolic causes. Cholecystokinin receptor-A (CCK-AR) is known to modulate satiety signal and delay gastric emptying, which are associated with FD. *CCK-AR* (rs1800857, T/C) polymorphism is associated with a defective splicing of the primary transcript of *CCK-AR* mRNA, which may result in the lower expression of the *CCK-AR*. Therefore, we evaluated the role of genetic polymorphism of *CCK-AR* gene (rs1800857, T/C) in FD.

## Material and methods

237 consecutive patients with FD (Rome III) and 250 healthy controls (HC) were genotyped for ***CCK-AR*** gene polymorphism (PCR-RFLP). Patients with FD were sub-classified into epigastric pain syndrome (EPS), post-prandial distress syndrome (PDS) and EPSPDS overlap.

## Results

Patients with FD [173 (73%) male, age 38±12-y] were comparable with HC [195 (78%) male, age 37±12-y] with respect to age and gender. 26/237 (11%) had EPS, 55 (23.2%) PDS and 156 (65.8%) EPSPDS overlap. Among 237 patients with FD, CC (variant) genotype of *CCK-AR* (rs1800857) was infrequent among patients than HC [19 (8%) vs. 46 (18.4%) p=0.001, odds ratio (OR) =0.36, 95% confidence interval (CI) =0.19-0.66]. However, genotypes distribution was comparable among patients with different subtypes of FD (p=0.44).

## Conclusions

CC genotype of *CCK-AR* polymorphism is protective for FD. EPSPDS overlap was common among patients with FD.

